# Is diagnosing patients with Organic Acidurias and Aminoacidopathies enough? Conundrums of a low middle-income country

**DOI:** 10.12669/pjms.37.7.3887

**Published:** 2021

**Authors:** Hafsa Majid, Lena Jafri, Zeba Zulfiqar Ali, Bushra Afroze

**Affiliations:** 1 Dr. Hafsa Majid, FCPS. Section of Chemical Pathology, Department of Pathology and Laboratory Medicine, Aga Khan University, Stadium Road, P.O. Box 3500. Karachi 74800, Pakistan; 2 Dr. Lena Jafri, FCPS, Section of Chemical Pathology, Department of Pathology and Laboratory Medicine, Aga Khan University, Stadium Road, P.O. Box 3500. Karachi 74800, Pakistan; 3 Zeba Zulfiqar Ali, Clinical Nurse Coordinator, Department of Paediatrics & Child Health. Aga Khan University, Stadium Road, P.O. Box 3500. Karachi 74800, Pakistan; 4 Dr. Bushra Afroze, FCPS, Department of Paediatrics & Child Health, Aga Khan University, Stadium Road, P.O. Box 3500. Karachi 74800, Pakistan

**Keywords:** Inherited metabolic disorder, Pakistan, Survey, Treatment, non-prescription, non-affordability, food for special medical purposes

## Abstract

**Objective:**

This study was done to determine the factors responsible for non-treatment of inherited metabolic disorders (IMDs) requiring food for special medical purposes (FSMPs) in Pakistan.

**Methods:**

A descriptive cross-sectional study was conducted by Departments of Pediatrics & Child Health and Pathology & Laboratory Medicine, Aga Khan University. Patients diagnosed with IMDs from January 2013 to December 2016 requiring FSMPs were surveyed after a year of initial diagnosis to collect the details of treatment advised, mortality status, and reasons of non-treatment, including not prescribed by physician, non-acceptance by family, non-availability or non-affordability.

**Results:**

Over four years period, 311 patients were identified with IMDs; Median age of patients was 1.0 yrs (0.0.2-3.65) with 54% (n=168) being male. Of the total 38.2% (n=119) required FSMPs, 9% (n=28) patients were excluded due to unavailability of diagnostics information. Parents of 58 patients requiring FSMPs out of 119 participated in survey. The leading causes of non-treatment were, FSMPs not prescribed by physicians (n= 30, 51.7%) followed by non-affordability (n=23, 39.6%), families’ unacceptance in (n=9, 18%) patients, non-availability of FSMPs (n=2, 3.4%) and early death of patient (n=1, 1.7%).

**Conclusion:**

The main factors responsible for non-treatment of FSMPs requiring IMDs were non-prescription by physician and non-affordability.

## INTRODUCTION

Inherited metabolic disorders (IMDs) encompass a heterogeneous group of genetic diseases due to defective metabolic processes. As defined recently, any condition in which the impairment of a biochemical pathway is intrinsic to the pathophysiology of the disease is considered an IMD.^1^ The cumulative incidence of IMDs varies between one in 1400 and one in 3000.^2^,^3^ The measured prevalence in a particular country depends on the population selected and the method employed for screening. The prevalence of IMDs is unknown in Pakistan but is expected to be high due to the cultural preference of consanguineous marriages.^4^,^5^

The IMDs caused by deficiency of enzymes in a metabolic pathway result in accumulation of toxic metabolites and deficiency of essential needed metabolites. Effective treatment of a group of IMD depends primarily on dietary restriction because off-the-shelf foods cannot be metabolized by these patients, resulting in toxic effects.^6^ Typically, a healthcare provider prescribes a specialized diet restricting the offending metabolites that cannot be metabolized while monitoring and providing all the other necessary metabolites essential for normal growth and development.^4^ For example, individuals with phenylketonuria are unable to properly metabolize the amino acid phenylalanine, which must be selectively limited in their diet to prevent severe intellectual disability.^7^,^8^ Disorders of amino acid and fatty acid metabolism require medical foods that restrict the offending amino acid(s) or long-chain fatty acids.^9^-^11^

Several organic acidemias (OA) and aminoacidopathies are treatable with the good outcome by Food for Special Medical Purposes (FSMPs), which is a life-long necessity for a patient.^12^,^13^ In 2013, through concerted efforts, FSPMs were made available locally. The local literature on the patient’s outcome diagnosed with IMDs, although limited, reports the poor outcome of these patients. Local studies have reported that many of the patients diagnosed with IMDs are not on treatment. There is a need to identify the issues leading to the non-treatment of these patients, design and implement strategies to overcome these issues and improve patient outcomes.

This study was conducted to obtain an up-to-date estimate of the outcomes of patients with IMDs requiring FSMPs treatment in the Pakistani population and determine the factors responsible for non-treatment of IMDs requiring FSMPs.

## METHODS

This descriptive cross-sectional study was conducted by the Departments of Pediatrics and Child Health and Pathology & Laboratory Medicine. Data of patients diagnosed with organic acidemias and aminoacidopathies based on urine organic acid and plasma amino acid testing from January 2013 to December 2016 were included in this study using a non-probability consecutive sampling technique. The treating physicians in different cities were informed of the diagnosis by telephone or email by a Chemical Pathologist about their patients’ diagnosis. Only organic acidurias and aminoacidopathies requiring FSMPs were included in this study, while those requiring orphan drugs for treatment were excluded. The fatty acid oxidation defects were not included in the present study, as they cannot be diagnosed on urine organic and amino acids analysis.

### Grouping of Inherited Metabolic Disorders

Organic acidemias and aminoacidopathies were categorized into two groups; *Decompensation Group*, which present with metabolic decompensation and encephalopathy and may lead to death when untreated. These include methylmalonic academia (MMA), urea cycle disorders (UCD), maple syrup urine disease (MSUD), Isovaleric academia (IVA), propionic acidemia (PA), glutaric aciduria type 1 (GA-1). The second group, the *Non-Decompensation group*, does not present with metabolic decompensation. The clinical course is rather static with intellectual disability and neurological impairment rather than mortality if not treated properly. These include phenylketonuria (PKU) & cystathionine beta-synthase (CBS) deficiency. Since it was a telephonic survey, intellectual disability and neurological impairment was not included as it requires formal assessment using structured age-appropriate tools by a trained clinical psychologist or neuro-developmental specialist.

### Survey to identify factors leading to treatment non-initiation

Only patients diagnosed with either organic acidemia or amino acidopathies for more than one year were included, and the survey was prospectively conducted from Jan to March 2018. Parents or guardian of patients were surveyed via telephone by the metabolic nurse in 10 to 15 minutes. Metabolic nurses are specialized clinical nurse coordinators trained in dealing with patients with IMDs, who have been working with a metabolic physician for the last four years. The principal investigator reviewed data collected by the metabolic nurse to identify missing data, and parents/guardian were contacted again to gather that missed data.

Details of treatment advised by the primary physicians, mortality status, and treatment not initiated or discontinued, reasons of non-treatment including not prescribed by the physician, non-acceptance by family, non-availability of FSMPs or non-affordability were collected.

### Outcome assessment of patients with decompensation group of IMDs

Patients included in the decompensation group of IMDs were also surveyed to gather data about their outcome in the form of mortality. Patients were categorized into two groups based on the healthcare provider’s speciality, i.e., under the care of a general pediatrician (practising all over Pakistan) or metabolic physicians (housed at AKUH only).

### Ethical Consideration

The study was done per Helsinki’s ethical code. In order to maintain confidentiality, coding was given to patients, and their original identifications were removed. An exemption was sought from Institution’s Ethical Review Committee (ERC number: Ped-5144)

### Statistical Analysis

The statistical analysis was performed using the statistical package social sciences version 19. Frequency and percentages were generated for categorical variables and median with interquartile ranges (IQR) for quantitative variables. A Chi-square test was performed to compare the outcome of patients under the care of general pediatricians and metabolic physicians.

## RESULTS

**Fig.1:**
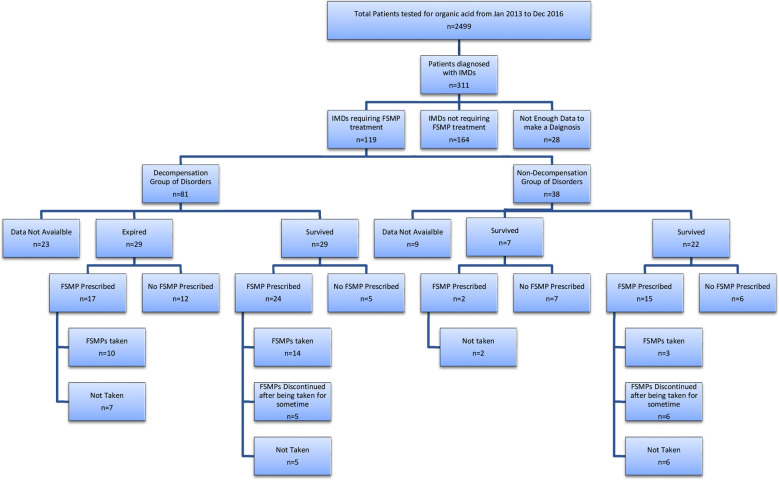
Distribution of study patients based on treatment status

Patients tested for OA and aminoacidopathies during the study period were 2499. Total 311 patients were tested positive for IMD. Median (Q3-Q1) age of patients was one years (0.0.2-3.65) with 54% (n=168) being male. Of the total 38.2% (n=119 of 311) required FSMPs for treatment, 52.7% (n=164) were in the non-FSMP treated group and 9% (n=28) patients were excluded due to non-availability of complete clinical and diagnostic information. The treatment status of patients is shown in Fig.1.

### Grouping of Inherited Metabolic Disorders

In the FSMPs treatment group, 68% (n=81 of 119) belonged to the decompensation group of IMDs, and 31.9% (n=38 of 119) were in the non-decompensation group. In the decompensation group, 34.4% (n=41 of 119) were prescribed FSMPs, and 20.1% (n=24of 119) of these took it, while the rest either did not take or discontinued FSMPs after some time, Fig.1. In the non-decompensation IMD group 14.3% (n=17) patients, were prescribed FSMPs, of which 2.5% (n=3) actually took it.

### Survey to identify factors leading to treatment non-initiation or discontinuation

**Fig.2:**
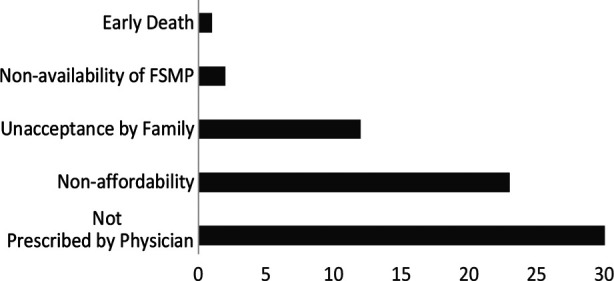
Distribution of factors leading to treatment non-initiation or discontinuation The number of responses is greater than the number of respondents because respondents were allowed to choose more than one response.

Data analysis was done using the Chi-square test to identify factors leading to non-initiation or discontinuation. Both alive and deceased patients who were prescribed FSMPs treatment were included in this survey; 48.7% (n=58 out of 119) patients. Top three leading causes for the treatment non-initiation or discontinuation were; FSMPs not prescribed by physicians in 51.7% (n=30) cases followed by non-affordability 39.6% (n=23) and families unacceptance in 18% (n=9), shown in Fig.2.

### Outcome assessment of patients with decompensation group of IMDs

Out of the total 119 patients that required FSMP, 68% (n=81 out of 119) had decompensated group IMD. Of the patients with a decompensated group of IMDs, 38% (n=31 out of 81) were under the care of a metabolic physician, while general pediatricians from all over Pakistan followed 62% (n=50 out of 81). Complete information was available for 29 patients in each group. Mortality was higher in patients under the care of general pediatricians than a metabolic physician, 62% (n=18/29 patient expired) and 38% (n=11/29 patient expired). The p-value determined using the chi-square test was 0.0572. While survival was better in patients treated by metabolic physician, 62% (n=18/ 29) vs. 38% (n=11/29), with a p-value of 0.0572.

## DISCUSSION

The prevalence of IMDs in our population is high.^14^-^17^ There is limited local literature available on different IMDs, which are mostly prevalence studies, but none have reported the outcome of IMD patients after diagnosis.^14^-^16^,^18^ Awareness regarding prompt diagnosis and early treatment of IMDs is increasing locally. Nevertheless, nearly half the IMD patients diagnosed are not on treatment. Therefore, there is a dire need to identify barrier in IMD patient’s management and reduce them. For IMDs requiring FSMPs, dietary therapy is the effective primary treatment. This is the first survey from Pakistan to obtain information about management and factors leading to the non-treatment of IMDs requiring FSMPs. In the present study, only one fifth (22.6%, n=27 of 119) patients requiring FSMPs were on appropriate treatment. Similar findings were reported by another study done from our centre during 2008-2012, which reported that only one-third of IMD patients were treated and under regular follow-up. Non-affordability was the main factor for non-compliance, as at that time, the FSMPs were imported in small batches at a high cost.^19^ The proportion of treatment non-compliance/non-initiation in the present study is almost the same as in the previous study, 51% vs 57%, respectively.

In the present study, non-prescription by the physicians was one of the major factors for treatment non-initiation. This point towards limited knowledge of general pediatricians regarding the appropriate use of FSMPs. These findings advocate the need for a knowledge assessment survey of the physicians taking care of IMD patients and better system integration. A local study on MMA patients reports similar finding for optimal outcomes.^20^ However, the sustainability of treatment is only possible if the FSMPs cost is more acceptable to families and donors. So, in order of significance, addressing the issue of high FSMPs cost needs to be prioritized. Similar findings were reported by a cross-sectional study done on Pakistani patients with Hyperphenylalaninemia, stating financial constraints as the major cause of treatment non-compliance.^21^

Healthcare infrastructure in Pakistan is very meagre. There is no health insurance system in Pakistan to cover the cost of FSMPs. The private sector provides health care services to almost two-thirds of the population, and health expenses are mostly paid out-of-pocket.^22^ The FSMPs are highly regulated, and specialized life-saving products used to treat IMDs, which are manufactured under strict medical vigilance, thus having a generally higher cost than standard infant formulas. Unfortunately, the FSMPs are not produced locally but are exported from other countries. However, the critical difference between the FSMPs as the lifesaving treatment and the regular infant formulas as food supplements is not recognized by the Government of Pakistan. Thus, FSMPs in Pakistan are imported under the same Harmonized System code (HS code 2016.9.9) as regular infant formulas. As a result, an accumulative tax of 70% is applied to this life-saving treatment.

**Fig.3:**
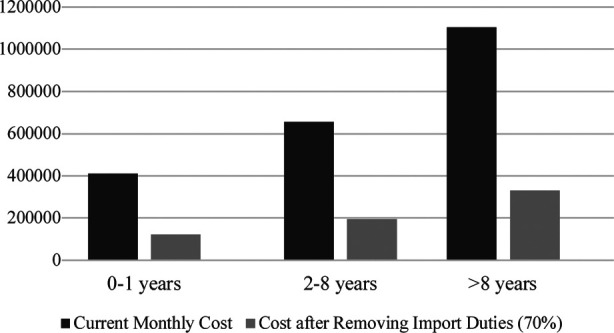
Estimated Annual Treatment Cost (PKR) of FSMP for MSUD in Pakistan, before and after removing the custom duties.

In contrast, the tax duties charged on importing FSMPs in countries like Iran and Turkey (HS Code 3004.5000) are 15% each. Due to the current tax structure, philanthropic agencies hesitate to support FSMP treatment as most philanthropic groups wish to transfer the full benefit of every penny donated directly to those in need of the support rather than paying the taxes to governments. Advocacy at the government level to reduce the tax on the FSMPs is critical for sustainable treatment. The impact of high import duties on FSMPs becomes evident when, for example, the treatment cost of MSUD is compared before and after applying custom duties in different age groups (Fig.2). If customs duties are removed, nearly 3.3 patients can be treated with the same amount of money currently spend to treat one patient, as shown in Fig.3.

In western countries, the situation is better due to medical insurance coverage, where medical insurances or other resources either completely or partially covers FSMPs cost.^23^ Locally published literature also reports that the high cost of FSMPs is a major cause of the low treatment compliance of patients.^24^ The high tax on the lifesaving FSMPs needs to be addressed in Pakistan for the patients diagnosed through high-risk screening and to pave the path of the newborn screen for IMDs in Pakistan, which is now the standard of care for all babies born in most of the World.

In the current study, only decompensation group patients were included as the patients with a non-decompensation group of IMDs develop intellectual disability with static clinical course and mortality was measured as the main outcome measure. Patients under the care of general pediatricians had a higher mortality rate than the patients under care of metabolic physicians. This points to the significance of specialized supervision of medical care of IMD patients given by the metabolic physician.

Another gap that needs to be addressed is to develop a good referral system for these patients. In Pakistan, seeking consultation from a specialist physician is mostly the parent’s responsibility. Moreover, a lack of timely referral to a metabolic specialist is also an important factor in poor patient outcome. An integrated referral system needs to be developed in Pakistan, and general pediatricians should be educated to use that system appropriately.

Families’ reluctance to accept the life-long treatment with various dietary restrictions and the use of FSMPs was also identified as a reason for non-compliance in our study. Many felt that they would be depriving their child of a normal life with life-long dietary restrictions. One patient was started on non-medical treatment in the form of homeopathic therapy. At the same time, two families gave up treatment only after a few weeks as they found no positive response, while one family did not initiate treatment due to their religious beliefs. These findings highlight the need to create public awareness of IMDs and the importance of long-term compliance to treatment. Public awareness activities may also include establishing patient-family support group, as engaging with families facing similar situations can help parents of newly diagnosed patients deal with the emotional stress of a chronic disease diagnosed in their child. These factors also highlight the importance of families being supported by a professional psychologist to help them cope with the emotional stress and challenges to deal with the life-long illness of their children. Families in Pakistan often do not have access to either of these support systems, mostly available in developed countries.

This is the first survey from Pakistan to obtain specific information of factors leading to non-treatment of IMDs patients requiring FSMPs. This study provides valuable information for those in policy planning regarding service provision for patients with IMDs, in Pakistan and similar populations.

### Limitations of the study

It includes a small sample size, and the call could not be recorded. Another limitation was that a telephonic survey and intellectual disability and neurological impairment assessment could not be done, requiring formal assessment using structured age-appropriate tools by a trained clinical psychologist or neuro-developmental specialist. Larger prospective, properly designed studies are needed to explore the findings of this study further.

## CONCLUSION

This survey identified that non-affordability and non-prescription were the main causes resulting in the non-treatment of IMDs patients requiring FSMPs. There is a dire need for policymakers and healthcare providers to work together to address these challenges for optimal outcomes.

### Authors Contribution:

**HM** collected, analyzed the data and prepared the manuscript.

**LJ** critically reviewed the manuscript for intellectual content.

**ZZA** conducted the telephonic survey, entered data and reviewed manuscript.

**BA** conceived the idea, supervised the project and critically reviewed manuscript for intellectual content and responsible for the accuracy or integrity of study.

All authors reviewed the final manuscript and agreed with its submission to PJMS.
